# A Novel Route for the Easy Production of Thermochromic VO_2_ Nanoparticles

**DOI:** 10.1002/chem.202102566

**Published:** 2021-11-05

**Authors:** Antonio J. Santos, Marta Escanciano, Alfonso Suárez‐Llorens, M. Pilar Yeste, Francisco M. Morales

**Affiliations:** ^1^ IMEYMAT: Institute of Research on Electron Microscopy and Materials University of Cadiz Puerto Real, Cádiz Spain; ^2^ Department of Materials Science Metallurgical Engineering and Inorganic Chemistry Faculty of Sciences University of Cádiz Puerto Real, Cádiz Spain; ^3^ Department of Statistics and Operative Investigation Faculty of Sciences University of Cádiz Puerto Real, Cádiz Spain; ^4^ INDESS: Institute of Research on Social and Sustainable Development University of Cádiz Jerez, Cádiz Spain

**Keywords:** differential scanning calorimetry, vanadium oxidation, vanadium dioxide synthesis, vanadium dioxide doping, X-ray diffraction

## Abstract

In this work, a simple, fast and dry method for the fabrication of a thermochromic product with a high load of VO_2_(M1) consisting of the controlled heat treatment of pure vanadium nanoparticles in air is presented. After a complete design of experiments, it is concluded that the most direct way to attain the maximum transformation of V into VO_2_(M1) consists of one cycle with a fast heating ramp of 42 °C s^−1^, followed by keeping 700 °C for 530–600 seconds, and a subsequent cooling at 0.05 °C s^−1^. Careful examination of these results lead to a second optimum, even more suitable for industrial production (quicker and less energy‐intensive because of its lower temperatures and shorter times), consisting of subjecting V to two consecutive cycles of temperatures and times (625 °C for 5 minutes) with similar preheating (42 °C s^−1^) but a much faster postcooling (∼ 8 °C s^−1^). These green reactions only use the power for heating a tube open to atmosphere and a vanadium precursor; without assistance of reactive gases or catalysts, and no special vacuum or pressure requirements. The best products present similar thermochromic properties but higher thermal stability than commercial VO_2_ particles. These methods can be combined with VO_2_ doping.

## Graphical abstract

**M1‐VO_2_ nanoparticles** with better thermochromic properties than commercial ones are fabricated by a simple, dry, fast, clean and safe method, compatible with VO_2_ doping, consisting of thermal treatments of V nanoparticles by fast heating and (a) keeping 700 °C for about 10 minutes and slow cooling (one cycle); or (b) 625 °C for 5 minutes and fast cooling (two cycles).

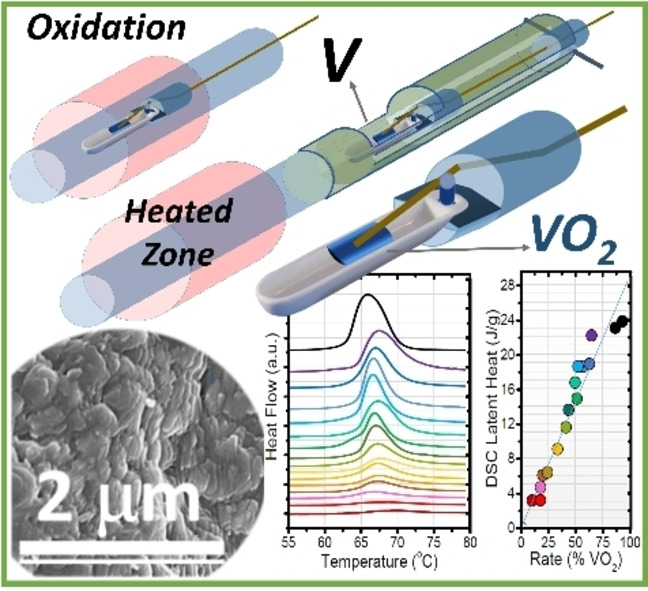

## Introduction

Vanadium is a transition element with an incomplete electronic structure and a consequent availability of multiple valences involving a complex and rich chemistry.^[1]^ Upon oxidation of V, the products can progress to mixtures of a great diversity of stoichiometries, and even into many crystalline varieties of the same vanadium oxide polymorph. For that matter, as shown in the different V‐O phase diagrams reported in the literature to date,[^2^, ^3^] the oxidation of vanadium leads to a wide variety of vanadium oxide compounds and phases which can coexist at temperatures below 1000 °C. In this context, the stable non‐equilibrium monoclinic dioxide VO_2_(M1) has received the greatest attention among these species since it transforms to rutile phase VO_2_(R) during heating, with an implicit reversible metal‐to‐insulator transition (MIT), which makes it the best candidate for applications in smart windows, switching electronics, heat storage, and other thermochromics’ uses.

Within this framework, nanoparticles have gained prominence in technological advances, since they offer the possibility of modulating and enhancing the physicochemical characteristics of materials for application in fields such as catalysis,^[4]^ optics,^[5]^ or medicine.[^6^, ^7^] Most of the trials attempting to synthesize a product of pure or of high load of VO_2_(M1) nanoparticles opted for gas‐phase or liquid‐phase reactions^[8]^ among which the hydrothermal path has been the most explored one because it permits a relatively good control of shape and crystallinity,[^9^, ^10^, ^11^, ^12^] although it is not the most scalable, cheap and fast way of production. Up to now, solid‐state reactions have been used for massive and low‐cost micron sizes of particles by thermal reduction of V_2_O_5_ in ammonia gas^[13]^ or oxalic acid,^[14]^ or by thermolysis.^[15]^ Nevertheless, these routes present some disadvantages: rigid experimental conditions, impurity of the rhombohedral shape product, and toxicity of the pentoxide for thermal reductions; and, for the thermolysis alternative: poor stability of the irregular shaped product, high aggregation of particles, and the use of a solution of liquid reactants to get a complex crystal precursor as well as organic solvents and surfactants to refine the particles. Dioxide nanoparticles with a mixture of M1 and M2 (a rare transitional polymorph also designed as “B”) were achieved by refluxing the pentoxide in an acidic solvent and a subsequent calcination,^[16]^ which is a time‐consuming process. Other approaches used for getting particles stacked on a substrate as the solution‐, the sputter‐, the gas phase‐, or the pulsed laser‐ methods also present associated drawbacks (see Table S1 of Ref. [17], for example).

On the other hand, layers of vanadium oxy‐nitride nanotubes were developed by wet electrochemical anodization of V sheets,^[18]^ while using O plasma to treat V strips promoted the formation of mixes of the dioxide and the pentoxide between 200 and 600 °C.^[19]^ It is worth noting the recent achievement of VO_2_ microtubes on a V_2_O_5_ substrate by a fast method of thermal oxidation of vanadium foils that needed a special heating source for reaching over 1700 °C.^[20]^ In an attempt to reach an economical and environmentally friendly solution on this matter, the present work describes a simple, fast, dry, cost‐effective, safe and clean method to get thermochromic VO_2_ particles from a metallic V precursor that is commercially available at a cheaper price than vanadium dioxide nanoparticles,^[21]^ and which can be obtained massively by economic routes, as for example, by high‐energy ball milling.^[22]^ Note that if needed for modulating the MIT temperature, this method is compatible with doping, for example by a preimpregnation of V with diluted cation salts (W, Mo, Nb, Ge.), and in this way, tungsten doping is demonstrated. Otherwise, this method can be applied to nanoporous films, dense thin films, or deposited nanoparticles of V.

## Results and Discussion

**Figure 1:**
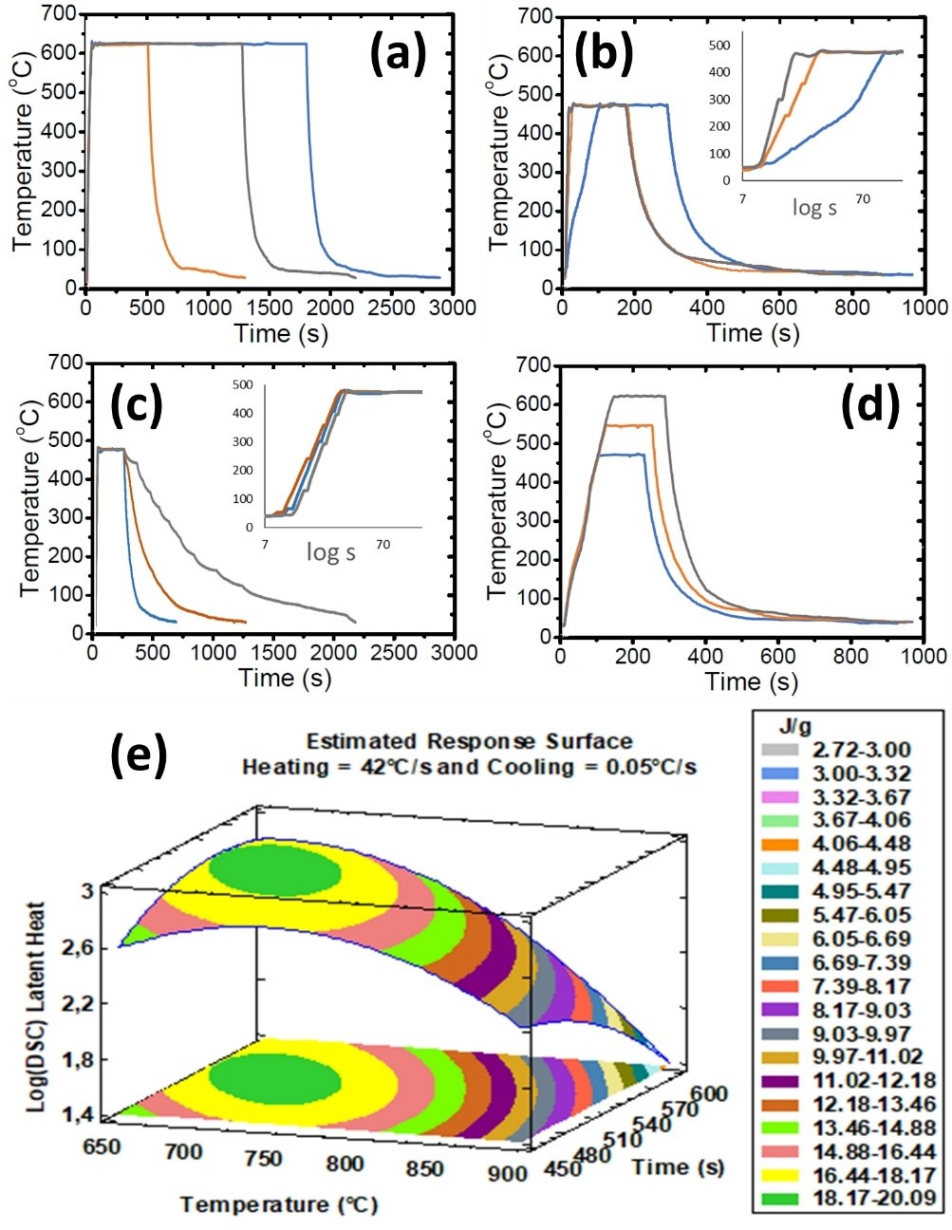
Temperature versus time tracks for representative thermal treatments: (a) fixed heating ramps of 17 °C s^−1^, maximum constant temperature of 625 °C for variable times of 400, 1200 and 1700 s, and fixed cooling rate of 0.6 °C s^−1^; (b) variable heating of 4, 17 and 42 °C s^−1^ till 475 °C during 150 s and cooling of 0.6 °C s^−1^; (c) heating of 17 °C s^−1^ till 475 °C for 180 s and variable cooling of 0.2, 0.4 and 0.6 °C s^−1^; (d) heating of 4 °C s^−1^ till varying temperatures of 475, 550 and 625 °C for 130 s and cooling of 0.6 °C s^−1^. (e) Fitting surface of optimum time versus temperature of the plateau, for fixed ramps of heating (42 °C s^−1^) and cooling (0.05 °C s^−1^), to achieve products with the bigger values of DSC latent heat of transformations. This is the result of the second phase of the DOE for a complete set of experiments.

The influence of key process parameters was verified by an exhaustive design of experiments (DOE). Tables S1, S2, S3 and S4 (Section A of the Supporting Information file) show the data associated to the 73 performed experiments resulting of programming (StatGraphics Centurion XVIII) first a randomized central composite design (CCD) model with four factors and three levels, and three further refinements to search for the optimum. We will denote by “A” the first group consisting of 30 randomized runs while improvements were named as series “B”, “C” and “D”. These tables collect the sample label A/B/C/D# for which # indicates the run order in the arbitrary sequence of experiments, the plateau temperatures (400 to 900 °C) and times (1 to 1000 s), plus the smaller or bigger average heating (4 to 42 °C s^−1^) and cooling rate (0.05 to 2.42 °C s^−1^) on each thermal recipe (the 4 factors with 3 varying values each), and the area under the endothermic DSC peaks at ∼67 °C, indicative of the enthalpy of the M1 to R phase transformation during heating. DOE was applied to determine the best conditions for obtaining the maximum transformation of V into VO_2_ given by the DSC area. Figure 1 (a–d) presents profiles for some exemplary thermal treatments to demonstrate the high control of experimental conditions applied.

It was concluded that the most direct way to obtain the maximum transformation of V into VO_2_(M1) consisted in one cycle with a fast heating ramp of about 42 °C s^−1^, followed by keeping a temperature around 700 °C for 530–600 seconds and a further slow cooling down at 0.05 °C s^−1^. Figure 1 e) summarizes that conclusion having a R^2^ of 90 %. Also, combinations of ranges of 750 to 900 °C with varying permanence times below 10 minutes are suitable to get almost similar results (average latent heat of the products of about 18 J g^−1^, similar to that of the as supplied commercial VO_2_), which is explained exhaustively in the Supplementary data (Section B of the Supporting Information file, Figure S1 to S3).

**Figure 2:**
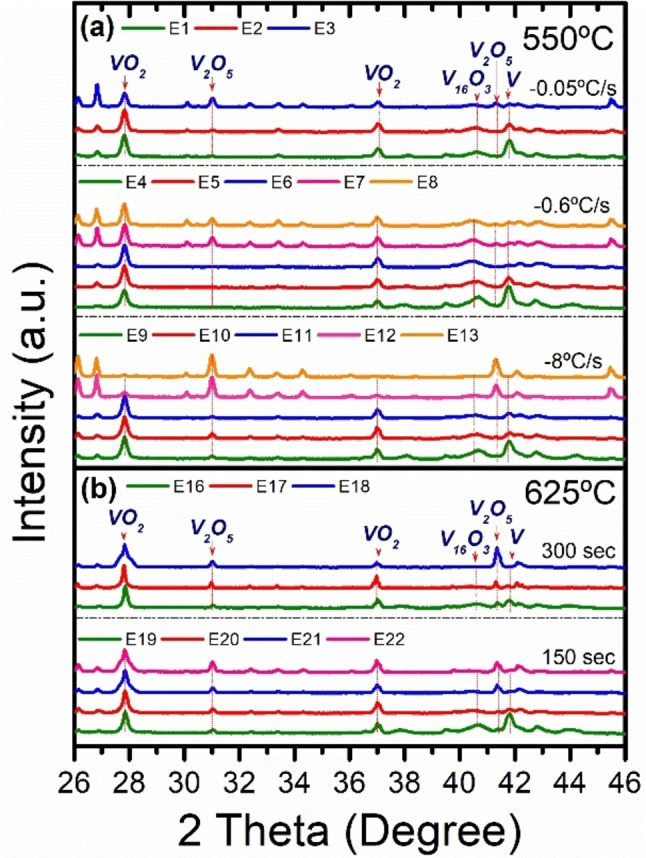
XRD diffractograms for different sets of thermal cycles. (a) Thermal cycles performed at 550 °C, for fixed reaction times of 300 seconds per cycle and heating rates of 42 °C s^−1^, using different cooling rates of 0.05, 0.6 and 8 °C s^−1^. (b) Thermal cycles performed at 625 °C, setting the heating and cooling rates at 42 °C s^−1^and 8 °C s^−1^, respectively, using reaction times of 300 and 150 seconds.

The examination of the diffractograms and calorimetry for the samples obtained in one cycle (series A to D) helped us to realize that for some experiments of lower maximum temperatures (i.e., A1, A2 or A4), there was an uncompleted consumption of the vanadium precursor, but the main oxide formed was the desired dioxide. This motivated new trials to check if consecutive oxidations could transform the remaining V into VO_2_, without transforming the dioxide portions previously formed. The XRD plots of most of these cycled samples (series E) are shown in Figure 2 (Table S5 also present their DSC data, in Section A of the Supporting Information file). Note that the best specimens of the consecutive rounds treated till 550 °C and varying cooling rates (E1 to E13), are E2 (2 cycles), E6 (3 cycles) and E11 (3 cycles). Unlike in the higher temperature regime, surprisingly, it is observed that increasing cooling velocities not only implies the need of more cycles to achieve better VO_2_ yields but also a cleaner VO_2_ product (see XRD peaks of Figure 2(a) and DSC latent heat values for E6 and E11). Provided that the fastest cooling rate worked better for the cycles till 550 °C, similar thermal cycles were conducted for maximum temperatures of 625 °C, varying, on this occasion, the reaction time of 300 or/and 150 seconds. In this regard, Figure 2 (b) and also the DSC values in Table S5, show that sample E17 gave the highest M1‐VO_2_ yields among the hundreds of oxidation products considered in the present study. Likewise, it was also proved that it was not possible to reach such good results by replicating the oxidation conditions of the E11 and E17 samples in a single stage (samples E14 and E23); this is to say, using reaction times equivalent to those resulting from the sum of the successive cycles, which brings to light the remarkable improvement achieved through the thermal cycling strategy. Additionally, thermal rounds were also performed at 475 °C and for 300 s (Table S5, samples E27–E28), but, as expected, the results were not prominent. Eventually, an alternative route based on mixed cycles was also explored, combining reaction times and temperatures (Table S5, samples E25 and E26), showing significant results that did not improve compared to the previous ones.

**Figure 3:**
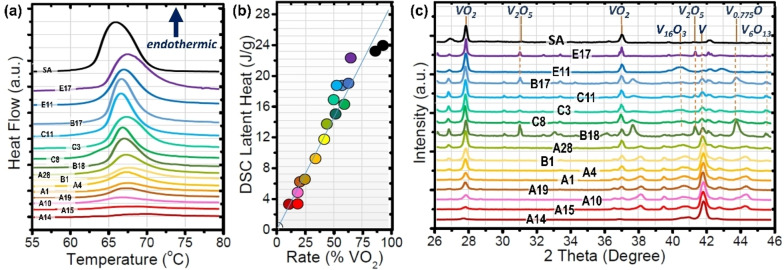
(a) DSC curves associated with the MIT on heating for the experimental conditions of oxidation and samples notation presented in the Supplementary data Tables S1–S5. (b) The direct linear relationship between the latent heat of transformation and the presence of the thermochromic phase in the end products of reaction. (c) XRD patterns for the same samples considered in (a).

We have supposed that the endothermic DSC peak areas, indicative of the enthalpy of the M1 to R phase transformation on heating, are associated with the amount of product transformed into M1‐VO_2_. Figure 3 demonstrates the adequateness of this assumption. Many samples with varying DSC values (left plot) were quantified by the Rietveld refinement (Section C of the Supporting Information file) applied to their XRD patterns (right plot). The central figure shows the linear relationship (R^2^=0.95) between the DSC value, and the percentage of thermochromic VO_2_, for 15 prototypical samples (labeled as in their own set of oxidations) in the full range of vanadium transformation rates, in addition to those similar results for the two commercial VO_2_ samples (black dots) and the V sample (white spot is obviously placed in the origin of coordinates because it is not thermochromic).

The right XRD compilation also gives an overview of the general dynamics of the studied system: there are two main stages in the progress of the oxidative reactions of one cycle, according to the majority of the phases present, the first one consists of the emerging formation of the dioxide by the consumption of the vanadium precursor till a level of saturation in which the second period begins. This advanced stage is characterized by a more extended oxidation of the V and the VO_2_, both acting as precursors for the formation of the tridecaoxide (sample C11); the pentoxide in combination with V_0.775_ O (sample B17); or these three oxides at the same time (sample B18). In general, it was found that there is a clear beneficial effect in decreasing the cooling rate, and that it is better when the heating rate is higher, although this is the least determining factor (it has a smaller influence in the response variable than the others). Nevertheless, there is a crossed effect between the maximum reaction temperature and the residence time at that temperature, and the best products come from finding confronted balances between both factors. In this sense, the highest DSC values (∼18,5 J g^−1^ in B17 or C11) for one cycle samples (M1 VO_2_ mainly combined with small amounts of V_2_O_5_ or V_6_O_13_) are at the level of many measurements for the portions of the 2 reference specimens used as supplied (SA1: 17–19 J g^−1^). But the experiments using cycles and fast cooling provided products with DSC values between 19 and 23 J g^−1^, and higher VO_2_ contents in combination with smaller quantities of V_16_O_3_ (E11) or of V_2_O_5_ plus V_6_O_13_ (E17), till matching the behavior of commercial samples refined in agate mortar (SA2: 22–24 J g^−1^), which is the same grind applied to the oxidized vanadium samples. Nevertheless, this commercial item has previously shown diverse values on the levels of the present studies, as for example, 16.2 J g^−1^,^[23]^ 17.8 J g^−1^,^[24]^ or 26.0 J g^−1^.^[25]^ A comprehensive description of all the crystalline phases of vanadium oxides that appeared, even if not cited in this paragraph, was carried out for all samples (Section C of the Supporting Information file).

**Figure 4:**
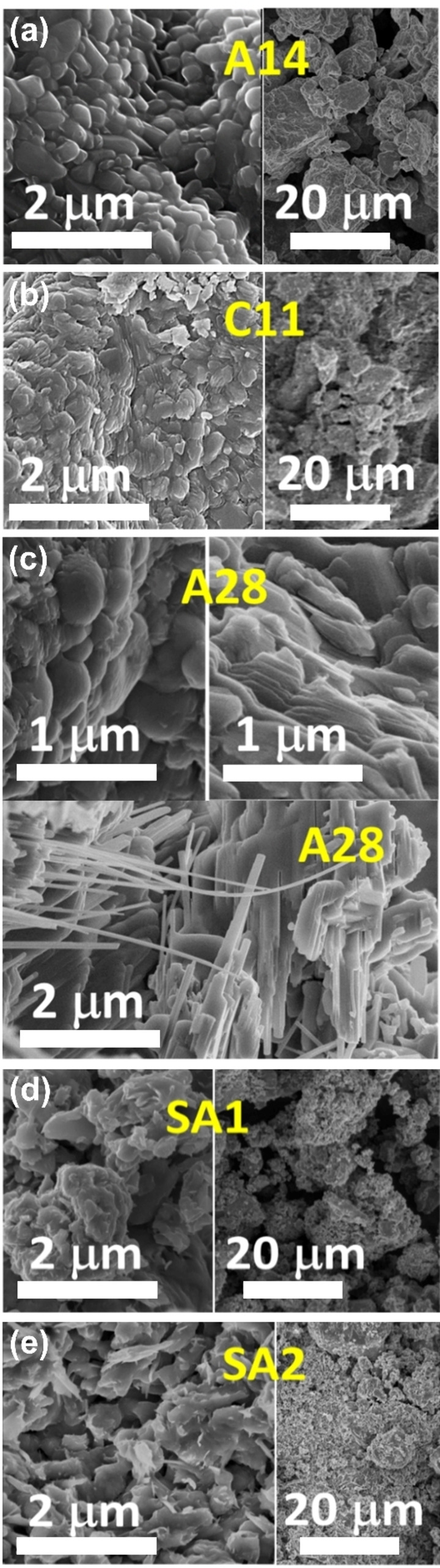
SEM micrographs of different stages of vanadium oxidation for the selected reaction products (a) A14, (b) C11, and (c) A28. (d and e) Images of the commercial VO_2_ used for comparison purposes.

Concerning the general features of the obtained products, it can be confirmed that the original vanadium nanoparticles that were oxidized as supplied (somehow agglomerated, with roughly spherical sizes of 100–200 nm in diameter), evolved to similar vanadium oxide aggregates in all samples, as shown in the examples imaged by SEM in Figure 4, representative of slightly (A14, bare product after reaction), or fully (C11, after hand milling), oxidized vanadium. However, other kind of textures (as nanotubes or facetted formations), were occasionally found in these samples (see examples for A28, archetypal of the moderate oxidation). Figure 4 also shows the aspect of the commercial VO_2_ sample as supplied (SA1) and after hand refinement (SA2). The characterization by TEM, including electron diffraction, of samples C11 and E17, demonstrates that M1‐VO_2_ is the main phase present in the more thermochromic samples (Section D of the Supporting Information file, including Figure S5 and S6).

**Figure 5:**
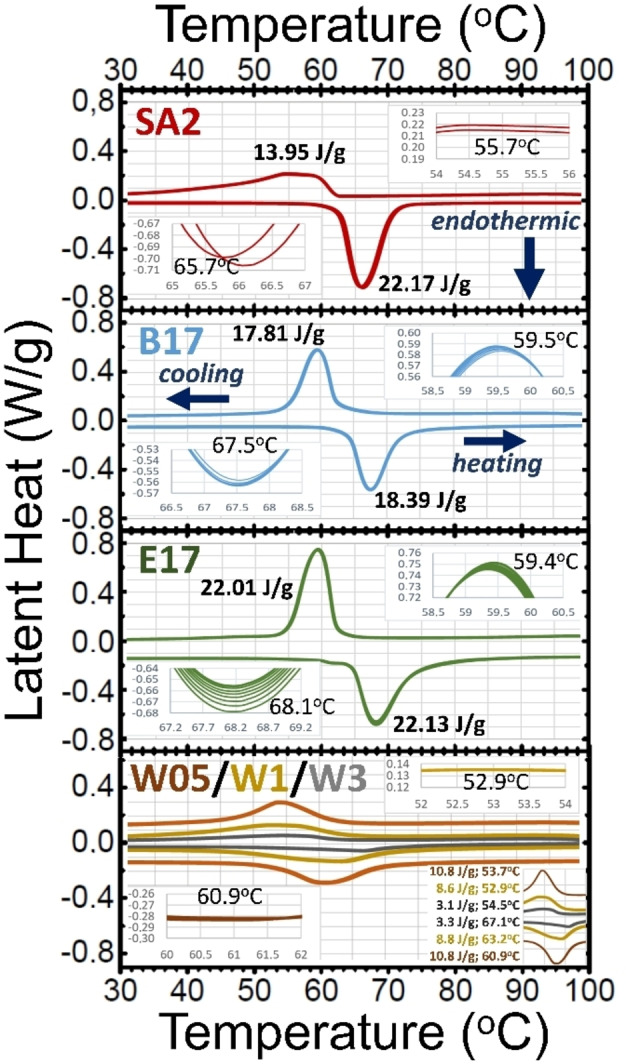
DSC plots for 2 sequences of heating and cooling for the VO_2_ standard (SA2); 10 sequences for two samples optimally thermally treated of one cycle (B17) and two cycles (E17); and 2 two sequences for oxidized powders impregnated with 0.5 (W05), 1 (W1) and 3 (W3) at.% of W with respect to V.

The proposed method also demonstrates that the thermochromic products have a good thermal stability and can be combined with doping of the VO_2_ particles in a simple way at the same time as they are synthesized. Figure 5 shows the DSC curves for 2 cycles of heating up plus cooling down for the sample SA2, for 10 similar cycles applied to samples B17 and E17, as well as for 2 of these cycles applied to 3 samples of oxidized V with increasing quantities of tungsten (W05, W1 and W3, respectively). These specimens received the same thermal treatment as sample C11. One surprising fact is that the latent heat of the commercial sample is clearly dissimilar for the transformations of heating and of cooling, both in the shapes and in the areas of the calorimetry peaks, with an exothermic enthalpy being half of the required energy for the endothermic phase change. A similar but not so apparent hint of asymmetry has been found for the same reagent.^[23]^ The positions of the minimum (at ∼67 °C) and maximum (at ∼62 °C) of the DSC plots (exo‐up) of samples B17 and E17 are in agreement with those of pure M1‐VO_2_,^[1]^ with similar areas (although more coincident for single‐cycle samples) under both peaks, this is a proof of the dioxide purity. In addition, the tracks of successive calorimetric scans of sample SA2 are not as coincident as those of the thermally treated products, even when they are mixed with W. This replication and high symmetry of the thermochromic hysteresis suggest a good thermal performance for potential applications. Note that for other thermochromic products obtained through thermal treatments of metastable VO_2_ phases, evident absence of replication of DSC curves appeared during successive modifications of temperature.^[11]^ On the other hand, the DSC plots for the tungsten preimpregnated powders show smaller values for both heating and cooling onsets (as expected[^26^, ^27^]), being this a demonstration of the effective doping. The decrease in the transition temperature and in the associated latent heat, however, are often a function of the dopant concentration;^[27]^ in our case, samples doped with smaller dopant concentrations not only lead to lower transition temperatures, but also to greater values of enthalpy (although still remarkably lower than those obtained for undoped products), which opens a new field of future research.

Concerning the overall quality of the results in the literature, some syntheses reaching VO_2_ products based on agglomeration of nanoparticles of different sizes by more complex ways than ours, have claimed similar latent heats near 67 °C for their optima in ranges from 15 till 25 J g^−1^.[^14^, ^20^, ^28^, ^29^, ^30^] Other investigations have shown that disaggregated particles with sizes from 24 to 46 nm can exhibit a higher degree of thermochromism (40 to 43 J g^−1^ showing an asymmetric MIT moved to 91–92 °C on heating^[29]^), but this disagrees with studies that found a trend of latent heats from 19 to 46 J g^−1^ for the endothermic peak at the expected position for pure dioxide when increasing the particles sizes from 10 to 55 nm,^[31]^ in accordance with calculations indicating a maximal performance for about 50 nm.^[32]^ Also, a recent patent claims particles with enthalpies of about 50 J g^−1^, but this observation is objectively unsupported.^[33]^ Note that this range (50‐60 J g^−1^) is a higher enthalpy value that is well accepted for single‐crystalline layers of VO_2_.^[34]^ We also have to emphasize that many studies support the goodness of their VO_2_ products on DSC plots, but they do not include enthalpy quantifications and just make qualitative comparisons based on graphs of heat flow with labelled or arbitrary units, or include quantitative values but supports on arbitrary unit plots.[^10^, ^11^, ^15^, ^24^, ^25^, ^27^, ^35^, ^36^, ^37^, ^38^, ^39^] Also, great care should be taken with literature results, for example, for the same Sigma‐Aldrich standard that was used here for comparison (89 and 94 % of M1‐VO_2_ according to XRD, ΔH∼23 J g^−1^) which often showed enthalpies below 26 J g^−1^ (reasonable for 99 % according to our central plot of Figure 3), two researches declared values of 31.2 J g^−1^ (on heating)^[16]^ or 38.7 J g^−1^ (on heating) and 42.1 J g^−1^ (on cooling)^[40]^ by DSC, and another one, estimated as 39.67 J g^−1[41]^ from thermogravimetric analyses. Without doubting the honesty of these scientists, when comparing their calorimetry curves to ours, and considering common masses of DSC measurements, it is hard to understand how for the same commercial product, sharp peaks reach, at the axis of W g^−1^, thrice (on heating) or even one order of magnitude (on cooling) higher than in our experiments. One might think that commercial batches could be heterogeneous, but bearing in mind the common strict quality control of the dealer, this issue might be due to possible miscalibrations of equipment.

It is thought that for polycrystalline VO_2_, the variations in the temperature of phase transition, decrease in its amplitude, and widening of the hysteresis width, happens due to lattice distortions at grain boundaries. However, the understanding of the influence on the thermal properties of particles of the type of processing, sizes and morphologies is not ever clear, since the rule of improvement by just miniaturizing and desegregating not always applies. For instance, induced lattice strains seem to have some key role, thus the doubling in the transition enthalpy was reported with annealing Sigma‐Aldrich VO_2_ standards in vacuum,^[23]^ or a decrease of intensity and a lowering of characteristic temperature was observed for the application of increasing pressures in VO_2_ sinters.^[42]^ In this latter cited work, a 180 micron available Japanese VO_2_ reagent was stated to have a ΔH value of ∼53 J g^−1^, and from another supplier of the same nationality,^[43]^ a substance with particle sizes of approximately 20 μm exhibited ΔH∼45 J g^−1^. Recently, another publication claims measurements of ΔH∼55 J g^−1^ for submicron and micron sized VO_2_ particles, and monotonic decreasing values till ΔH∼10 J g^−1^ as the particles synthesized by the same method become more and more miniaturized till 30 nm.^[38]^ A quantity of ΔH∼45 J g^−1^ was also reported for rhombohedral crystals of several tens of micrometers.^[13]^ Nevertheless, very different shaped and heighted heat flow peaks (plotted in arbitrary units) derive to a majority of similar latent heat values in this reference. It was observed too that microparticles behaved better than nanotubes for the same fabrication method.^[36]^ Certainly, there is some room for improvement based on the control of VO_2_ sizes, morphologies and pressures at the mesoscale, where our synthesis method, that has its own obvious technical advantages, can provide greater profits in the future.

## Conclusions

Through the analyses of more than 100 experiments of V oxidation, a simple, dry, fast, clean and safe method (it does not involve harmful, polluting or explosive reagents or (sub)products, and it is scalable to an industrial level for the manufacture of nanoparticles, either operating in continuous flow or batch processing) to fabricate thermochromic particles with a high load of VO_2_ and properties similar to those of commercial pure VO_2_, has been presented. Unlike previous attempts with the state of the art technology for synthesizing VO_2_ nanoparticles, the proposed reactions offer the advantage of being environmentally friendly and cheaper since they only use electricity to heat a furnace; without the assistance of special gases, liquids, or catalysts, neither vacuum, nor overpressure; and with the sole need of a precursor of V particles much more economical than VO_2_ particles. In this sense, through the accurate control of the reaction temperatures and times as well as the heating and cooling rates, two optimal thermal routes were achieved: (a) a single‐cycle which implies a heating ramp of about 42 °C s^−1^, reaction temperatures and times of about 700 °C and 530–600 seconds, respectively, and a cooling rate of 0.05 °C s^−1^; and (b) two consecutive thermal cycles at 625 °C and 300 seconds, with heating and cooling rates of 42 °C s^−1^and 8 °C s^−1^, respectively. Thanks to XRD, DSC, SEM and TEM studies, it was evidenced that the resulting products obtained by means of these two pathways, which were proved to contain high loads of the M1‐VO_2_ thermochromic phase, not only presented phase‐transition latent heat values of about 18 and 22 J g^−1^ for heating, which are comparable to those obtained for pure commercial VO_2_ particles, but also showed better thermal performances and stabilities than this standard VO_2_ reference. The doping susceptibility of the best products was subjected to study as well. In this regard, a simple approach based on the incipient wetness preimpregnation of vanadium powders with an aqueous solution of ammonium metatungstate hydrate was proved to be successful, attaining thermally stable W‐doped VO_2_ products. Finally, after a careful revision of previous works in this field, it is concluded that the outcomes achieved through this simple methodology are remarkable, but there is still a margin for improvement by controlling the VO_2_ sizes, morphologies and pressures at the mesoscale.

## Experimental Section

For each oxidation, pure vanadium with a BCC structure and spherical shape and size in the range of 100–200 nm (from Nanografi AS, ≥99.95 % of V) was used, organized as micrometric agglomerates of nanoparticles. Special care has been taken with the purity and crystalline nature of the precursor, since other dealers claiming to sell a similar article supplied a powder with a very high grade of impurities (i.e. Nanoshel LCC). Two VO_2_(M1) standards (Sigma‐Aldrich, ≥99 % trace metals basis) were also used for comparison of the transformation yield in our products of reaction. A characterization of commercial items and products was carried out by scanning electron microscopy (SEM), and by X‐ray diffraction (XRD) and fluorescence (XRF) (these latter data are not provided in this work).

**Figure 6:**
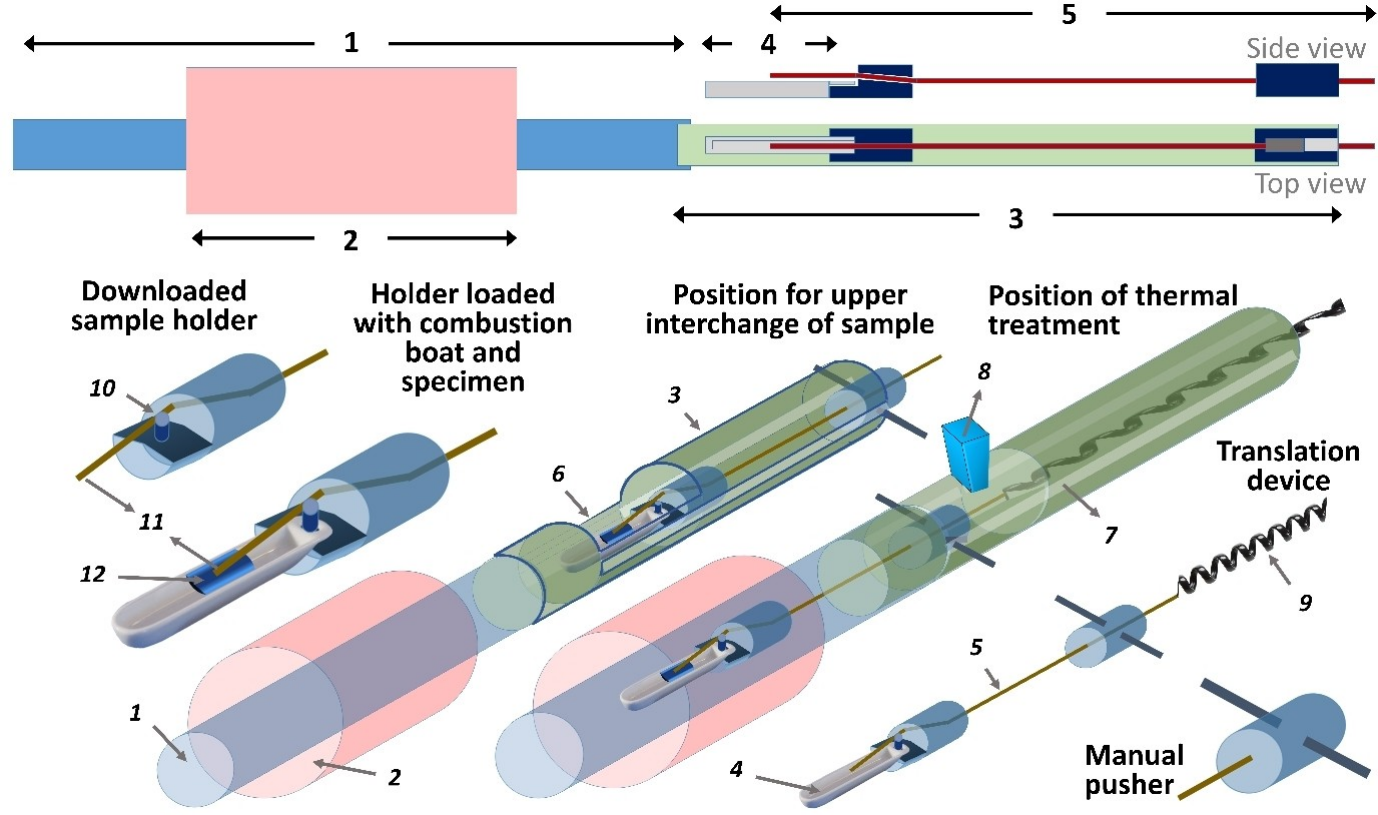
Schematic overview of the homemade reactor design, and details of all of its essential parts, used for the discontinuous flux thermal treatments of V nanoparticles to fabricate VO_2_. Arrows and numbers place emphasis on: 1) ceramic tube; 2) furnace; 3) metallic tube; 4) combustion boat; 5) thermocouple‐beam; 6) top window for load; 7) rail‐guide of translation; 8) cooling air intake; 9) thermometer signal wire; 10) hitch for reaction boat; 11) thermocouple tip; and 12) specimen.

The V nanoparticles were thermally treated in a homemade reactor of discontinuous flow (Figure 6) consisting of a ceramic tube inside a SiC resistors furnace able to reach 1500 °C, with an attached concentric metallic tube where a high‐temperature steel covered k‐type thermocouple inside it, acts as an axle for a system of horizontal translation. At the end of the metallic tube nearby the furnace, the thermocouple crosses and fixes to a cylinder placed inside this tube, mechanized with a hitch to hang a combustion boat. Thus, the thermometer tip is always placed some millimeters over the center of this alumina crucible (where a layer of vanadium with a mass of 140 mg is distributed in a width of 1 cm in the center of that boat), allowing the temperature in the reaction zone to be life‐tracked. The other end side also crosses and is fixed to another piece that is part of a handlebar used to slide the specimen holders inside and outside. When the boats are outside the furnace, they can be interchanged through an open window on top of the metal tube, which also has a cut guide for moving the translation device in and out. In this way, by fixing a set up temperature, it is not only possible to modulate the heating rate of the thermal treatment, but also to reach and stabilize certain temperature in the reaction zone (below the settled one) by moving the boat towards (heating) or outwards from (cooling) the center of the furnace (region of maximum temperature). A faster cooling than that reached on open air can be obtained by placing onto the top window a pipe blowing compressed air towards the hot specimen. Consequently, routines of translations were prepared for the chosen average heating and cooling velocities, or for longer or shorter residence times at a desired temperature.

In addition to SEM (FEI Nova NanoSEM) and XRD (Cu radiation Panalytical X'Pert PRO MPD, sometimes combined with Rietveld quantification), the techniques used for studies were differential scanning calorimetry (DSC) on TA Instruments Q20 and Q200 systems at standard conditions of heating and cooling (10 °C min^−1^) between 5 and 105 °C, and transmission electron microscopy (TEM) on Jeol 2100 and FEI Titan^3^ microscopes, for nanoparticles ultrasonicated for few minutes in a mixture of water and ethanol, and dropped onto a holey carbon TEM grid. In general, all particle samples were milled and well mixed into an agate mortar before their studies by DSC, XRD and TEM. The tungsten‐doped products were prepared by incipient wetness impregnation of vanadium in several steps with an aqueous solution of ammonium metatungstate hydrate (Sigma‐Aldrich, 99.99 % trace metals basis). After impregnation, the sample was dried in an oven at 105 °C for 24 h. The corresponding impregnation‐drying cycles were completed until obtaining homogeneous mixtures, with contents of 3 % (W3 sample); 1 % (W1); and 0.5 % (W05) atomic W, with respect to V.

## X‐ray crystallography

Deposition Number https://www.ccdc.cam.ac.uk/services/structures?id=doi:10.1002/chem.202102566 1924040 (for V), 1606741 (for VO_2_(M1)), 1612688 (for V_2_O_5_), 1638095 (for V_16_O_3_), 1604835 (for V_6_O_13_), and 1689621 (for V_0.775_O) contains the supplementary crystallographic data for this paper. These data are provided free of charge by the joint Cambridge Crystallographic Data Centre and Fachinformationszentrum Karlsruhe www.ccdc.cam.ac.uk/structures Access Structures service.

## Conflict of interest

The authors declare no conflict of interest.

## Supporting information

As a service to our authors and readers, this journal provides supporting information supplied by the authors. Such materials are peer reviewed and may be re‐organized for online delivery, but are not copy‐edited or typeset. Technical support issues arising from supporting information (other than missing files) should be addressed to the authors.

Supporting Information
