# Supplementary material for: A Novel Route for the Easy Production of Thermochromic VO2 Nanoparticles
Source: Chemistry. 2021 Nov 5;27(67):16662–9. doi: 10.1002/chem.202102566 (PMC9297899; doi:10.1002/chem.202102566)
Supplement: Supplementary file 1 — Supporting Information [file CHEM-27-16662-s001.pdf]

Chemistry–A European Journal

Supporting Information

A Novel Route for the Easy Production of Thermochromic VO_2 Nanoparticles

Antonio J. Santos, Marta Escanciano, Alfonso Suárez-Llorens, M. Pilar Yeste, and Francisco M. Morales*

Supporting Information

SECTION A. COLLECTION OF EXPERIMENTS OF VANADIUM OXIDATION

Table S1. Experiments performed in the first phase of the design.

Sample	Temparature, °C	Time, s	Heating, °C/s	Cooling, °C/s	DSC area, J/g
**A1**	550	150	23	0.4	5.98
**A2**	550	300	23	0.4	8.93
**A3**	625	1	4	0.2	9.52
**A4**	475	300	42	0.2	9.00
**A5**	550	150	23	0.6	6.71
**A6**	625	300	42	0.6	8.51
**A7**	625	300	4	0.6	6.15
**A8**	550	150	23	0.4	6.43
**A9**	550	150	23	0.4	7.26
**A10**	475	1	4	0.2	4.57
**A11**	625	1	42	0.2	9.03
**A12**	625	1	42	0.6	4.39
**A13**	550	150	23	0.4	5.92
**A14**	475	1	42	0.6	3.03
**A15**	475	1	42	0.2	3.11
**A16**	550	150	23	0.4	6.86
**A17**	550	150	23	0.4	6.80
**A18**	625	1	4	0.6	5.70
**A19**	550	150	42	0.4	6.32
**A20**	475	150	23	0.4	6.48
**A21**	625	150	23	0.4	7.76
**A22**	625	300	4	0.2	11.25
**A23**	550	150	23	0.2	8.18
**A24**	550	150	4	0.4	7.87
**A25**	550	1	23	0.4	3.86
**A26**	475	300	4	0.6	8.51
**A27**	475	300	42	0.6	8.35
**A28**	625	300	42	0.2	11.49
**A29**	475	1	4	0.6	3.57
**A30**	475	300	4	0.2	10.08

Table S2. Experiments performed in steps 1 and 2 of the second phase of the design.

Sample	Temparature, °C	Time, s	Heating, °C/s	Cooling, °C/s	DSC area, J/g
**B1**	550	300	42	2.42	7.98
**B2**	550	375	42	2.42	9.95
**B3**	550	450	42	2.42	9.83

**B4**	475	375	42	2.42	8.33
**B5**	475	450	42	2.42	8.02
**B6**	475	525	42	2.42	9.08
**B7**	400	450	42	2.42	3.97
**B8**	400	525	42	2.42	3.53
**B9**	400	600	42	2.42	6.34
**B10**	625	300	42	0.05	13.98
**B11**	625	375	42	0.05	14.15
**B12**	625	450	42	0.05	15.03
**B13**	700	375	42	0.05	15.04
**B14**	700	450	42	0.05	15.03
**B15**	700	525	42	0.05	16.34
**B16**	775	450	42	0.05	15.50
**B17**	775	525	42	0.05	18.54
**B18**	775	600	42	0.05	13.49
**B19**	625	525	42	0.05	14.60
**B20**	625	600	42	0.05	16.18
**B21**	625	675	42	0.05	16.18
**B22**	625	750	42	0.05	16.00
**B23**	700	532	42	0.05	16.40
**B24**	700	532	42	0.05	14.50

Table S3. Experiments performed in the third step of the second phase of the design.

Sample	Temparature. °C	Time. s	Heating. °C/s	Cooling. °C/s	DSC area. J/g
**C1**	775	525	42	0.05	15.91
**C2**	850	525	42	0.05	12.19
**C3**	700	525	42	0.05	16.67
**C4**	850	450	42	0.05	11.32
**C5**	775	600	42	0.05	10.82
**C6**	775	450	42	0.05	17.99
**C7**	700	450	42	0.05	15.94
**C8**	775	525	42	0.05	14.79
**C9**	850	600	42	0.05	7.007
**C10**	775	525	42	0.05	17.81
**C11**	700	600	42	0.05	18.51

Table S4. Experiments performed in the third phase of the design.

Sample	Temparature, °C	Time, s	Heating, °C/s	Cooling, °C/s	DSC area, J/g
**D1**	475	1000	42	0.05	11.37
**D2**	550	907	42	0.05	15.54
**D3**	850	254	42	0.05	17.39
**D4**	900	30	42	0.05	18.05
**D5**	725	300	42	0.05	15.06
**D6**	775	120	42	0.05	13.77
**D7**	830	30	42	0.05	14.86
**D8**	830	10	42	0.05	15.20

Table S5. Experiments performed to prove the goodness of sequential cycles of thermal treatments.

Sample	Temparature, °C	Time, s	Heating, °C/s	Cooling, °C/s	Number of cycles	DSC area, J/g
**E1**	550	300	42	0.05	1	12.27
**E2**	550	300	42	0.05	2	14.31
**E3**	550	300	42	0.05	3	9.29
**E4**	550	300	42	0.6	1	8.80
**E5**	550	300	42	0.6	2	12.77
**E6**	550	300	42	0.6	3	16.08
**E7**	550	300	42	0.6	4	10.89
**E8**	550	300	42	0.6	5	11.13
**E9**	550	300	42	8.0	1	12.11
**E10**	550	300	42	8.0	2	16.21
**E11**	550	300	42	8.0	3	18.80
**E12**	550	300	42	8.0	4	4.23
**E13**	550	300	42	8.0	5	1.59
**E14**	550	900	42	8.0	1	15.82
**E15**	550	1200	42	8.0	1	13.13
**E16**	625	300	42	8.0	1	15.68
**E17**	625	300	42	8.0	2	22.13
**E18**	625	300	42	8.0	3	19.28
**E19**	625	150	42	8.0	1	11.45
**E20**	625	150	42	8.0	2	17.50
**E21**	625	150	42	8.0	3	19.78
**E22**	625	150	42	8.0	4	17.75
**E23**	625	600	42	8.0	1	15.66
**E24**	625	900	42	8.0	1	14.54
**E25**	625/625	300/150	42	8.0	2	18.84
**E26**	625/550	300/300	42	8.0	2	20.18
**E27**	475	300	42	8.0	1	10.14
**E28**	475	300	42	8.0	2	8.19
**E29**	475	300	42	8.0	3	6.70

SECTION B. DESING OF EXPERIMENTS AND REFINEMENT FOR ONE-CYCLE OXIDATIONS

The Response Surface Methodology (RMS) was applied to determine the best conditions for obtaining the maximum transformation of V into VO_2 (given as the peak area of the DSC curves for the heating transformation of the products). RMS was performed employing the Statgraphics Centurion XVIII software. In order to plan and conduct the experiments, a strategy of experimentation based on several phases was followed.

The first phase of the experiment

In the early stages of the research and mainly based on both the literature and technical conditions we had just identified four potential significant factors. Due the fact we were able to address all factors a specific DOE screening technique was not necessary. In the first RMS phase, a central composite design (CCD) model with four factors and three levels consisting of 30 randomized runs with six replicas at the central points was used to optimize the process variables (maximum "temperature" of reaction (A), residence "time" at this temperature (B), "heating" rate (C) and "cooling" rate (D)). Table S1 (A1-A30) shows the independent factors and levels to design the response surface matrix, and also the experimental values of the response variable (Y: DSC area, or the latent heat of phase change on heating). In order to reduce the prediction uncertainty, we have removed the four least not significant terms from the initial model, namely, the quadratic terms AA, BB, CC and the interaction term BD, having p-values 0.4765, 0.2129, 0.5661 and 0.7347, respectively. Table S6 shows the ANOVA table of the final fitted model and the polynomial regression equation. Figure S1 shows the response surface depending on time (B) and temperature (A), and the standardized effect given by the Pareto diagram.

Table S6. Analysis of variance for Y

Source of Variation	Sum of Squares	df	Mean Square	F-test	P-Value
**Linear effect**					
A:Temperature	16.2469	1	16.2469	57.94	0.0006
B:Time	70.0139	1	70.0139	249.67	0.0000
C:Heating	0.885781	1	0.885781	3.16	0.1357
D:Cooling	25.2642	1	25.2642	90.09	0.0002
**Interaction effect**					
AB	10.3893	1	10.3893	37.05	0.0017
AC	1.01153	1	1.01153	3.61	0.1160
AD	10.9445	1	10.9445	39.03	0.0015
BC	1.66217	1	1.66217	5.93	0.0590
CD	0.615048	1	0.615048	2.19	0.1987
**Quadratic effect**					
DD	2.42695	1	2.42695	8.65	0.0322
**Error**					
Lack of fit	5.21837	14	0.37274	1.33	0.4019
Pure error	1.40214	5	0.280428		
Total (corr.)	146.081	29			
**Polynomial regression equation**					
Y = -12.27 + 0.04148A + 0.05011B – 0.1464C + 11.6D-0.00007187AB+ 0.0001764AC – 0.05514AD + 0.0001135BC+ 0.0516CD + 14.51DD					

The final model has a R^2 of 95.4679%. The estimate of the residual standard deviation is 0.529554. The Durbin-Watson test (p-value = 0.8143) shows no autocorrelation in the residuals at a significant level 0.05. Finally, the lack-of–fit test of goodness (p-value =0.4019) also shows the model fits well the data at the 95% confidence level.

Conclusion of the first phase. The analysis of variance (Table S6) indicates that the response variable depends on the linear terms of the processing variables temperature (A), time (B) and cooling (D), the interaction terms AD and AB, and the quadratic term DD (p-value < 0.05). It also shows a slight dependency on the interaction term BC. In order to interpret the surface response, we compute in Table S7 the partial derivatives of the polynomial regression equation. First, it is clear that there is not a stationary point in the experimental range. Second, just observing the sign of the partial derivatives in the experimental region, the response variable increases when time increases and cooling decreases. Therefore, the optimal solution is achieved at time=300s and cooling=0.2°C/s. Third, conditioning to those values the response variable always increases when temperature increases and then taking temperature=625°C, we finally obtain that the response variable also increases when heating increases. In conclusion, the optimal solution is achieved at a vertex of the experimental region at A=625°C, B=300s, C=42°C/s, and D=0.2°C/s. The optimal response value is 11.5696 J/g. Due to the fact that the global optimum seems to be out of the experimental region, some additional data are required. We next provide some remarks which will help us to move our process variables.

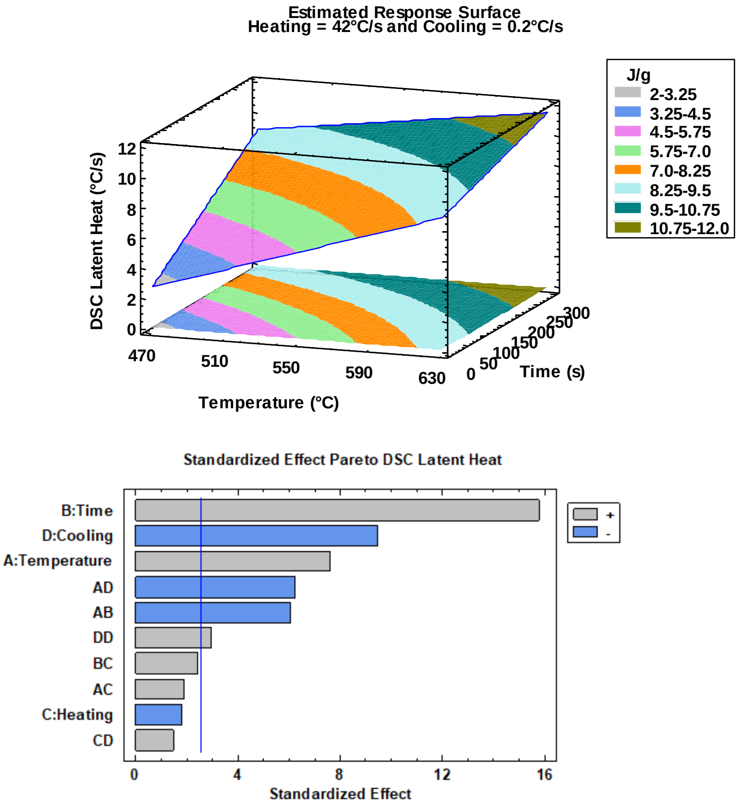

Figure S1. Response surface depending on A (Temperature) and B (Time) and Pareto diagram.

Table S7. Range of values of the partial derivatives in the experimental region.

Partial derivative	Equation	Range of values in the experimental region
∂Y/∂B	0.05011-0.00007187A+0.0001135C	0.00564525 ≤ ∂Y/∂B ≤ 0.02073875
∂Y/∂D	11.6-0.05514A+0.0516C+14.51D	-18.3756 ≤ ∂Y/∂D ≤ -3.7183
∂Y/∂A	0.04148-0.00007187B+0.0001764C-0.05514D	B=300s. D=0.2°C/s<br>0.0095966 ≤ ∂Y/∂A ≤ 0.0162998
∂Y/∂C	-0.1464+0.0001764A+0.0001135B+0.0516D	A=625°C. B=300s and D=0.2°C/s<br>∂Y/∂C = 0.00822

Remark 1. Figure S2 shows a direct extrapolation of the response surface out of the experimental region. Although in the experimental region the response variable always increases when time and temperature increase, the negative sign of the interaction term AB suggests that for a certain threshold the effect of time on the response variable is opposite depending on the value of temperature and vice versa. Just fixing heating = 42°C/s and cooling = 0.2°C/s a straightforward computation shows the response surface has a theoretical saddle point at temperature =763.56 °C and time = 526.79s out of the experimental region. A saddle point is neither a maximum nor a minimum for the surface response. The existence of that saddle point suggests the response will start decrease when temperature and time exceed a theoretical threshold. Additionally, it should be explored the effect on the response when time decreases and temperature increases and vice versa.

Remark 2. Although in the experimental region the response variable always decreases when cooling decreases, having a higher cooling value opens up a new path of investigation. Just observing the partial derivatives given in Table S7, a higher value of cooling has a negative impact in the partial derivative of the temperature. This fact suggests that fixing a large value of cooling we could expect the response variable increases when temperature decreases.

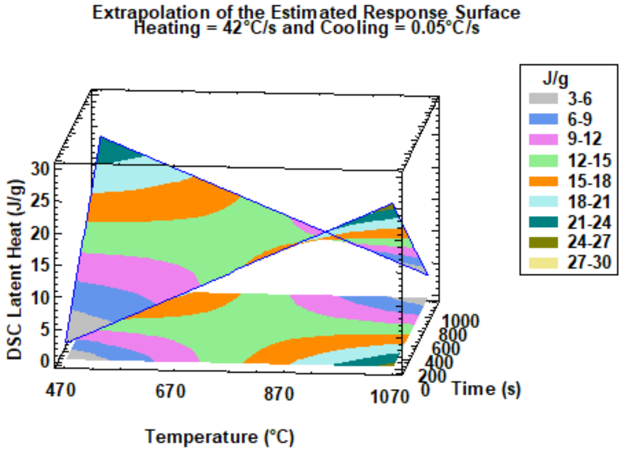

Figure S2. Extrapolation of the response surface out of the experimental region of phase 1.

The second phase of the experiment

In order to explore the different research paths, we split the second phase in several steps.

Step 1. In light of Remark 2, the effect of having a large value of cooling was first explored. Due to the available technical conditions, D=2.42°C/s was fixed. By also fixing C=42°C/s, we theoretically expect the response variable increases when time increases and temperature decreases. Table S2 shows nine new runs (B1-B9) consisting in decreasing the temperature and increasing the time. It is apparent that these data do not reflect the hypothesis assumption. Therefore, we decided to close this research path.

Step 2. Here, a theoretical saddle point is looked for, by increasing simultaneously time and temperature. It was noticed in the first phase the response variable in the experimental region increases when cooling decreases, having the cooling the second major standardized effect. Therefore. we decide to fix the cooling process variable to the minimum feasible technical value. i.e., to 0.05°C/s. Using a similar argument, the heating process variable was also fixed to the maximum feasible technical value. i.e., to 42°C/s. Now taking in account Remark 1, nine new runs were carried out to study the effect on the response variable of simultaneously increasing time and temperature (B10-B18 of Table S2). It was first empirically observed that the response has larger values compared to those in the first phase. Second, the response keeps increasing

when time and temperature increase. Finally,. we also empirically observe how the response begins to decrease at A=775ºC and B=600s which reinforces the existence of a saddle point. All those runs help us to move our process towards a local growth direction. Note that runs B19-B24 were performed with the purpose of validation.

Step 3. Here, the process is moved to confirm the existence of a theoretical saddle point. By fixing heating = 42ºC/s and cooling =0.05ºC/s, a full factorial design (FFD) model with two factors and three levels consisting of 12 randomized runs with three replicas at the central point was used to optimize the process variables (temperature (A) and time (B)). Table S3 (samples C1-C11) shows the independent factors and levels to design the response surface matrix and also the experimental values of the response variable (Y). One of the replica at the central point was taken from B17 in the previous step. As the error (residuals) seems to be larger in this phase, we decide to use the logarithmic transformation of the response. Table S8 shows the ANOVA table of the final fitted model and the polynomial regression equation. Figure S3 shows the response surface depending on time and temperature (complementary to Figure 2(e)), and the standardized effect given by the Pareto diagram. The final model has a R^2 of 88.22%. The estimate of the residual standard deviation –in logarithmic scale- is 0.103547. The Durbin-Watson test (p-value = 0.2543) shows no autocorrelation in the residuals at a significant level 0.05. Finally, the lack-of–fit test of goodness (p-value =0.2380) also shows the model fits well the data at the 95% confidence level.

Conclusion of the second phase. The analysis of variance (Table S8) indicates that the response variable depends on the linear terms of temperature (A) and time (B). It also shows a slight dependency on the interaction term AB and the quadratic term BB. The mathematical optimal solution is achieved inside the experimental region at A=700ºC and B=529.083s. The optimal response value is Y = exp(2.94123) = 18.94 J/g. The polynomial regression equation obtained at this stage reinforces the result pointed out in Remark 1. It can be observed how the response starts to decrease when temperature and time exceeds a theoretical threshold. It is also apparent that there exists a region where the response variable does not vary significantly. It is worth noticing that the residual standard deviation provides a larger uncertainty compared to that obtained in the first phase of the experiment, σ=exp(0.103547)=1.11 J/g. To conclude, in Table S9 a validation of this surface is provided by comparing the adjusted and observed value for two observations close to the experimental region (B19,B20), two other observations more distant of this experimental region (B21,B22) and, finally, two other ones inside the experimental region (B23, B24).

Table S8. Analysis of variance for Log(Y). Heating = 42°C/s and Cooling = 0.05°C/s.

Source of Variation	Sum of Squares	df	Mean Square	F-test	P-Value
**Linear effect**					
A:Temperature	0.441002	1	0.441002	41.13	0.0077
B:Time	0.117209	1	0.117209	10.93	0.0455
**Interaction effect**					
AB	0.0989546	1	0.0989546	9.23	0.0560
**Quadratic effect**					
AA	0.0517024	1	0.0517024	4.82	0.1156
BB	0.0689076	1	0.0689076	6.43	0.0850
**Error**					
Lack of fit	0.079641	3	0.026547	2.48	0.2380
Pure error	0.032166	3	0.010722		
Total (corr.)	0.949425	11			
**Polynomial regression equation**					
Log(Y) = -27.5329 + 0.04943A + 0.04981B – 0.00002796*AB – 0.00002475*A^2 – 0.00002857*B^2					

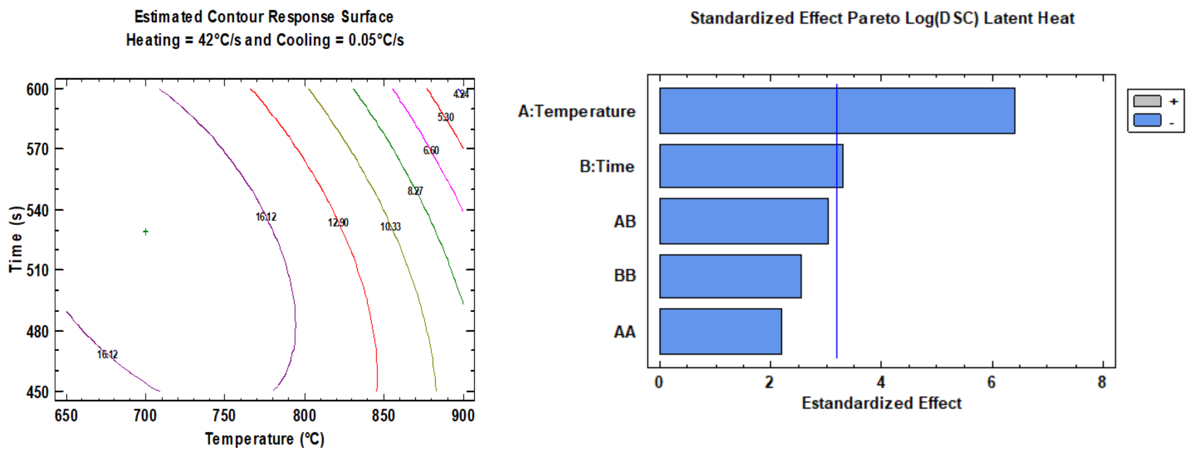

Figure S3. Response surface out of the experimental region after phase 2.

Table S9. Validation of the fitting surface by comparing adjusted and observed values for two observations close to the experimental region, and other two farer of this experimental region.

Temperature	Time	Adjusted	Lower Prediction Limit	Upper Prediction Limit	Log(Observed)
625	525	2.79413	1.93009	3.65818	2.68102153
625	600	2.80819	1.92601	3.69037	2.75174806
625	675	2,50074	1.33373	3.66776	2.78377591
625	750	1.8718	-0.119084	3.86268	2.77258872
700	532	2.94099	2.53748	3.34449	2.79728133
700	532	2.94099	2.53748	3.34449	2.67414864

The third phase of the experiment

Based on the extrapolation given in Figure S3 it is natural to explore the effect of the pairs high temperature and short time and vice versa. For such a purpose we carried out new eight runs given in Table S4 (samples D1-D8). Note that those experimental values were considered by starting from a hypothetical center and the technical extreme values. Data reflect that the response is far from the optimum value when temperature decreases and time increases and it takes values close to the optimum one when temperature increases and time noticeably decreases.

By considering all experimental values obtained through the different phases having heating = 42 °C/s and cooling = 0.05 °C/s, i.e., collecting data from samples B10-B24, C1-C11 and D1-D8, we compute a R^2 of 61.03%. We first empirically observe that variability increases when temperature increases and time decreases. We second note that the optimal values obtained in the second phase could also be reached as a combination of high temperatures, 830-900°C, and time values less than 250 seconds. In fact, the sample D4 (900°C and 30 s) provided 18.05 J/g. Nevertheless, we think a new experiment should be designed to study in depth the effect of the response variable when temperature and time noticeably increases and decreases, respectively. On the other hand, higher process temperatures to reach similar yields Iess less attractive for a possible transfer of the synthesis method to industry.

Final conclusion

By starting at temperature= 475°C and time = 1s the response variable increases when temperature and time increase simultaneously. However, there exists a theoretical point where this effect is the opposite. i.e., the response starts to decrease when time and temperature increases. Additionally, for low temperatures the response variable increases when time increase but it seems those response values are far from the ones obtained by increasing simultaneously time and temperature. On the other hand, for high temperatures the response variable increases when time decreases obtaining response values close to the optimum. In this way, the following paragraph was stated in the article, concerning the supplementary sections A and B:

The influence of key process parameters was verified by an exhaustive design of experiments (DOE). Tables S1, S2, S3 and S4 (Section A of the Supporting Information file) show the data associated to the 73 performed experiments resulting of programming (StatGraphics Centurion XVIII) first a randomized central composite design (CCD) model with four factors and three levels, and three further refinements for the search of the optima. We will denote by "A" the first group consisting of 30 randomized runs while improvements were named as series "B", "C" and "D". These tables collect the sample label A/B/C/D# for which # indicates the run order in the arbitrary sequence of experiments, the plateau temperatures (400 to 900°C) and times (1 to 1000 s), plus the smaller or bigger average velocity of the heating (4 to 42°C/s) and cooling (0.05 to 2.42°C/s) on each thermal recipe (the 4 factors with 3 varying values each), and the area under the endothermic DSC peaks at ~67°C, indicative of the enthalpy of the M1 to R phase transformation during heating. DOE was applied to determine the best conditions for obtaining the maximum transformation of V into VO_2 measured as the DSC area. Figure 2 (a-d) presents profiles for some exemplary thermal treatments to demonstrate the high control of experimental conditions applied. It was concluded that the more direct way to attain the maximum transformation of V into VO_2(M1) consisted in one cycle of a fast heating ramp of about 42°C/s, followed by keeping a temperature around 700°C for 530-600 seconds and a further slow cooling down at a velocity of 0.05°C/s. Figure 2 (f) summarizes that conclusion having a R^2 of 90%. Also, combinations of ranges of 750 to 900°C with varying permanence times below 10 minutes are suitable to get almost similar results (average latent heat of the products of about 18 J/g, similar to that of the as supplied commercial VO_2), which is explained in a whole extension in the supplementary data (Section B of the Supporting Information file, Figure S1 to S3).

SECTION C. X-RAY DIFFRACTION: IMPLICATED PHASES AND RIETVELD QUANTIFICATIONS

The XRD diffractograms recorded throughout the series A, B and C (single cycle) revealed the unwanted formation of different vanadium oxides. In this sense, it is essential to find out which oxidation conditions promote the upwelling or decreasing of such oxides in order to limit their generation and thus achieving a purer VO_2 final product. Figure S4(a)-(d) display the progression of the characteristic XRD peaks of many of the resulting oxides in accordance with the oxidation conditions. A preliminary overview of these graphics lead to discern between oxides generated at severe conditions of temperature and reaction times (V_2O_5, V_6O_{13} and V_2O_3) and other oxides whose development occurs at moderate temperatures and reaction times (VO, $V_{16}O_3$ and VO_x).

With regard to the oxides formed at severe conditions, V_2O_5 (Figure S4(a)) and V_2O_3 (Figire S4(c)) species stand out, which start to emerge from 700ºC and 775ºC respectively, becoming more extensive as reaction time increases. The arising of these two species always leads to a decrease in the generated VO_2, which suggests that the dioxide can act as one of the reagents for the formation of the trioxide and the pentoxide. Hence, limiting the formation of these two oxides becomes a key issue in the search for high VO_2 yields. In this way, it should be also highlighted the formation V_6O_{13} at temperatures above 625ºC (Figure S4(b)). However, it seems that its generation is stopped at about 700ºC, probably due to the preferential development of V_2O_5 and V_2O_3.

Figure S4(d) shows the progression of VO, $V_{16}O_3$ and VO_x, the latter being considered as a mixture of metastable and intermediate vanadium oxides. As can be observed, the signals of all these oxides become weaker for greater temperatures and times, which may imply that they are first oxides to be formed, even earlier than the VO_2 itself. As a point of interest, it is worth mentioning the progressive evolution of VO into $V_{0.775}O$ at higher temperatures (above 625ºC) and reactions times (beyond 300 seconds), leading to a more oxygen enriched specie (O/V ratio = 1.29). In this light, it is thought that VO, $V_{16}O_3$ and VO_x could act as seeds for the formation of VO_2 when the temperature is kept below 625ºC since, as mentioned above, higher temperatures would promote the development of V_2O_5, V_2O_3 and V_6O_{13} species with the subsequent VO_2 consumption.

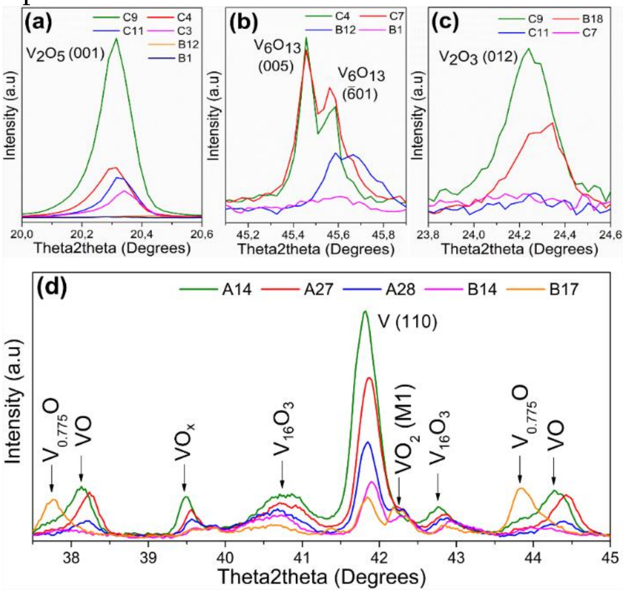

Fig S4. Details of XRD spectra for many vanadium thermally treated specimens illustrating the various phases appearing at the different analyzed oxidation stages.

On the other hand, all the XRD quantitative analyses were carried out by the Rietveld [*H. M. Rietveld, A profile refinement method for nuclear and magnetic structures. J Appl Cryst 2 (1969) 65-71*] method with X'Pert Highscore Plus software from PANalytical B.V., version 4.8. The refined overall parameters were: cell parameters, zero-shift error, peak shape parameter and phase fractions. Background functions were accounted for by selecting "use available" tool of the software. Peak shapes were fitted by using the pseudo-Voigt function [*P. Thompson, D.E. Cox, J.B. Hasting, Rietveld refinement of Debye-Scherrer synchrotron X-ray data from Al2O3, J. Appl. Crystallogr. 20 (1987) 79-83*] with the asymmetry correction included [*L.W. Finger, D.E. Cox, A.P. Jephcoat, A correction for powder diffraction peak asymmetry due to diaxial divergence, J. Appl. Crystallogr. 27 (1994) 892-900*] by refining U, V and W (Gaussian contribution) and peak shape 1 (Lorentzian contribution) when appropriated. Crystal structure descriptions for all the phases were those published in [*M. A. G. Aranda, A. G. De la Torre, L. Leon-Reina, "Rietveld quantitative phase analysis of OPC Clinkers, Cements and Hydration products" in "Applied mineralogy of cement and concrete" Eds. Jodi. J. Rosso, Mineralogical society of america. 2012 ISBN: 978-0-939950-88-1*]. When the Rietveld scale factors are directly transformed to phase contents, RQPA gives the mineralogical composition neglecting the non-diffracting content, and, therefore, referred to 100% of crystalline computed material, so the amorphous content is not take it into account.

As can be seen in Table S11, XRD quantifications disclosed the presence of minority amounts of the VO_2(M2) polymorph, which also presents thermochromic properties, especially in samples belonging to the serie A of experiments. Note that, throughout this work, all the references made to the thermochromic VO_2 phase have been only referred to the VO_2 M1 phase, always neglecting the M2 polymorph. Nonetheless, it is also worth mentioning that the sum of M1 and M2 phases gives rise to similar linear relationships between the DSC value, and the percentage of these two thermochromic VO_2 phases (R^2=0.95). Likewise, metastable and intermediate vanadium oxides (VO_x) were found in samples of the A series as well. In this way, due to the limited information available in crystallographic databases for such species, they were finally indexed as "impurities" in the Highscore Plus software. Finally, it is also worth mentioning that, although they were quantified by the Rietveld method as well, the samples E2 and E6 were not included in the studies referred in Figure 4 of the article.

Table S11. Rietveld quantitative phase analysis for different samples.

Sample	Composition (wt%)
**A1**	22.9% VO; 21.8% VO_x; **20.0% VO_2(M1)**; 18.3% $V_{16}O_3$; 13.4% V; 3.5%VO_2 (M2)
**A4**	**34.1% VO_2(M1)**; 20.4% $V_{16}O_3$; 15.4% V; 14.2% VO; 14.2 VO_x; 1.7%VO_2 (M2)
**A10**	31.9% VO; 19.2 VO_x; **18.1% VO_2(M1)**; 17.1% $V_{16}O_3$; 12.7% V; 0.9%VO_2 (M2)
**A14**	28.0% VO_x; 25.8% VO; 18.2% V; 16.5% $V_{16}O_3$; **10.3% VO_2(M1)**; 1.2%VO_2 (M2)
**A15**	62.0% V; 19.8% $V_{16}O_3$; **18.2% VO_2(M1)**
**A19**	25.3% $V_{16}O_3$; **24.6% VO_2(M1)**; 17.8 VO_x; 17.7% VO; 14.6% V
**A28**	**41.6% VO_2(M1)**; 17.9% $V_{16}O_3$; 17.2% V; 9.0 VO_x; 7.7% VO; 6.5%V_6O_{13}
**B17**	**57.7% VO_2(M1)**; 16.5% $V_{0.775}O$; 13.9% V_6O_{13}; 8.1% V_2O_5; 3.8% V
**B18**	**44.1% VO_2(M1)**; 28.7% $V_{0.775}O$; 10.5% V_2O_5; 8.3% V_6O_{13}; 3.4% V; 2.9% V_2O_3; 1.9% $H_{7.24}O_{13}V_6$
**C3**	**50.0% VO_2(M1)**; 17.6% V_6O_{13}; 12.3% V_2O_5; 9.6% $V_{16}O_3$; 5.9% V; 4.6% VO
**C8**	**51.7% VO_2(M1)**; 16.3% VO; 10.8% V_2O_5; 9.3% V_6O_{13}; 5.4% $V_{16}O_3$; 4.7% V; 1.9% V_2O_3
**C11**	**52.9% VO_2(M1)**; 13.1% V_2O_5; 10.1% V_6O_{13}; 9.3% VO; 6.8% V; 6.0% $V_{16}O_3$; 1.4% V_7O_4; 0.4% $H_5O_8V_3$
**E2**	**49.4% VO_2(M1)**; 16.7% V_6O_{13}; 13.7% V_2O_5; 13.0% $V_{16}O_3$; 7.2% V
**E6**	**59.5% VO_2(M1)**; 31.1% $V_{16}O_3$; 5.5% V_2O_5; 3.9% V_6O_{13}
**E11**	**63.3% VO_2(M1)**; 12.3% $V_{16}O_3$; 10.9% V_2O_5; 7.4% V_6O_{13}; 5.1% V; 1.1%VO_2 (M2)
**E17**	**65.2% VO_2(M1)**; 30.2% V_2O_5; 7.4% V_6O_{13}; 3.9% $V_{16}O_3$; 0.7% V
**SA1**	**87.2% VO_2(M1)**; 7.7% V_2O_3; 5.1% $Cr_{0.2}O_2V_{0.8}$
**SA2**	**93.9% VO_2 (M1)**; 6.1% V_2O_3

SECTION D. TRANSMISSION ELECTRON MICROSCOPY STUDIES OF OPTIMAL SPECIMENS

High-resolution TEM (HRTEM) and selected area electron diffraction (SAED) were performed for freestanding isolated or aggregates of nanoparticles in C11 and E17, in order to ratify the presence of monoclinic VO_2 proved by XRD as the majoritarian phase of their complete mass. Many reflections at SAED patterns could be indexed for M1-VO_2 (not shown here), along with lattice distances in HRTEM micrographs. Figure S5 shows a collection of irregular shape particles detached from aggregates of sample C11 after ultrasonication, and HRTEM images of two different particles, with insets showing atomic columns of monoclinic VO_2 (M1) and of a V_6O_{13} single crystals, both oriented in the [001] zone axis, with families of planes and their parameters and angles highlighted. Figure S6 shows three HRTEM images of three different regions within the same agglomeration of particles from sample E17, which exhibit lattice resolution proper from the thermochromic VO_2 (M1) phase or orthorhombic V_2O_5 as demonstrated by the indexed families of planes and angles.

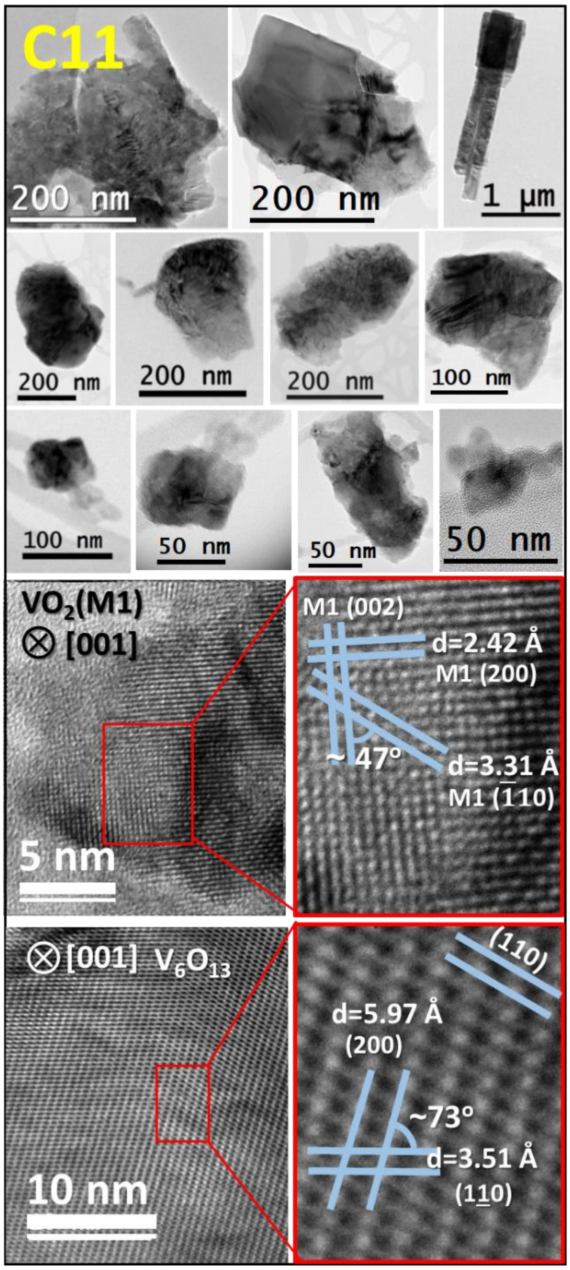

Figure S5. TEM studies of sample C11.

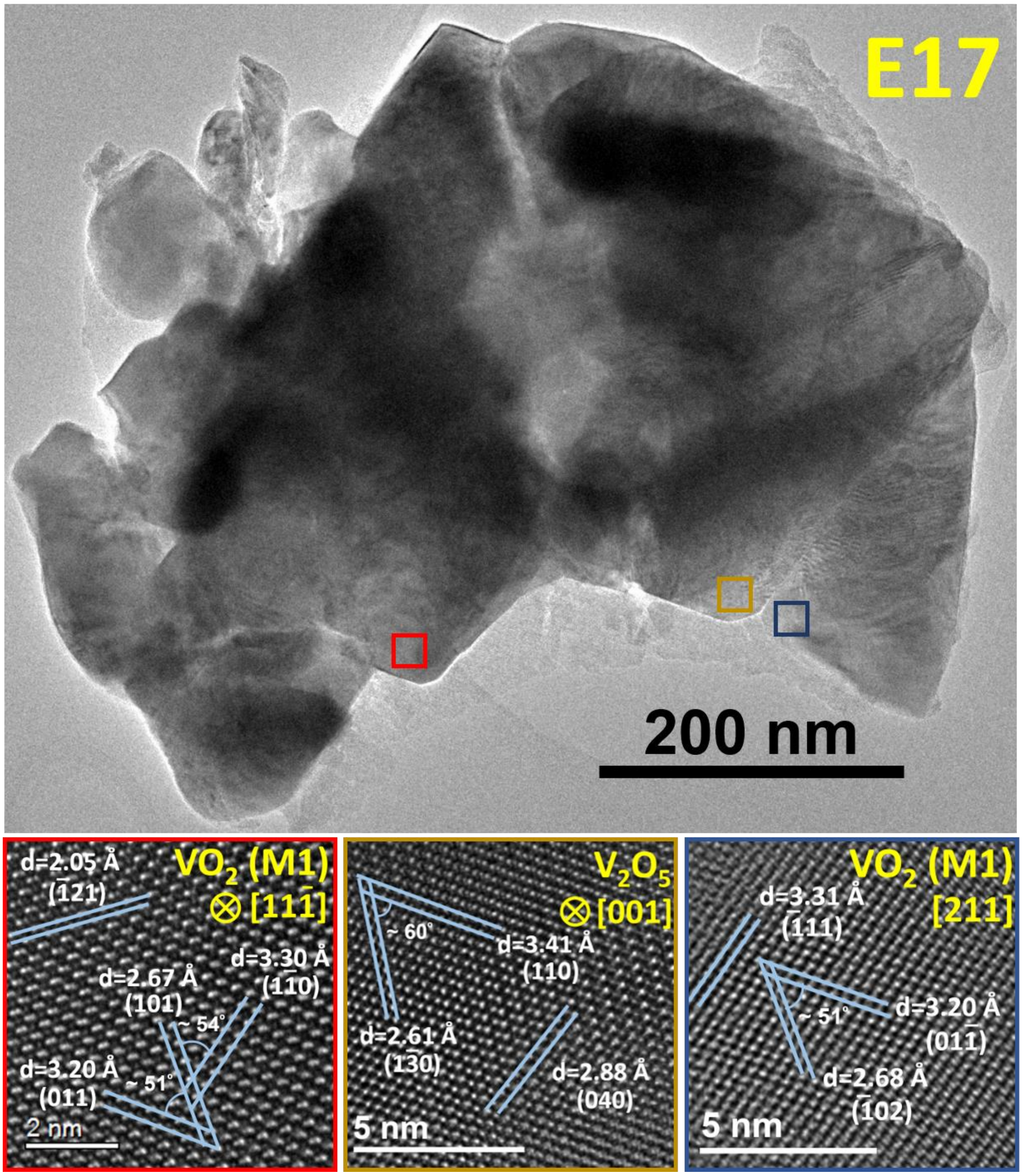

Figure S6. TEM studies of sample E17.
